# Long-term outcome of incidental cystic liver tumors in the general population

**DOI:** 10.1038/s41598-021-91140-3

**Published:** 2021-06-03

**Authors:** S. F. U. Blum, T. Ittermann, M. L. Kromrey, C. M. Dreyer, D. Seppelt, R. T. Hoffmann, H. Völzke, J. P. Kühn

**Affiliations:** 1 Institute and Polyclinic for Diagnostic and Interventional Radiology, University Hospital Carl Gustav Carus, Technical University Dresden, Fetscherstraße 74, 01307 Dresden, Germany; 2 Institute of Community Medicine, University Medicine Greifswald, Greifswald, Germany; 3 Institute of Diagnostic Radiology and Neuroradiology, University Medicine Greifswald, Greifswald, Germany

**Keywords:** Liver, Liver diseases, Endocrine system and metabolic diseases

## Abstract

Aim of this study was to investigate frequency, incidence and risk factors of liver cysts in the general population in a longitudinal survey. Cyst frequency was investigated in 607 adult volunteers (288 women, 319 men, mean age 55 years) using strong T2-weighted magnetic resonance imaging. Risk factors were investigated for occurrence, frequency and size of cystic lesions at baseline. Incidence and physiological growing of the lesions were observed in a 5-years follow-up. At baseline, 431 volunteers had 1,479 cysts (71.0%). The mean number of cysts per person was 3.4 ± 9.0. The mean size of cysts was 13.1 ± 11.7 mm. Women had a higher number of cysts than men (*p* = 0.026). Older and male volunteers demonstrated a higher cyst frequency (*p* = 0.002 and *p* = 0.025). Per one-year increase in age the chance for a liver cyst increased by 2%. Four-hundred seventeen volunteers had cysts in the follow-up, in 24.6% new lesions had occurred. Lesion size significantly increased in follow-up (*p* < 0.001). Age and male sex were associated with the occurrence of at least one liver cyst. Women had a higher average number of cysts. Cystic lesion progression is a physiological phenomenon in the long-term follow-up.

## Introduction

Cystic hepatic lesions are a common incidental finding^1–4^. They can occur as solitary or multiple lesions. Unfortunately, cystic hepatic lesions have many differential diagnoses^4–6^. In adults benign cystic hepatic lesions are divided into developmental (simple cysts, biliary hamartomas, Caroli disease), inflammatory (abscesses, hydatid cysts), and trauma-related lesions (biloma)^2,4,6^. Most simple hepatic cysts are of congenital origin, but some of them can develop during life.

Cross-sectional imaging such as computed tomography and magnetic resonance imaging (MRI) helps to differentiate the dignity of cystic liver lesions^3^. MRI provides a high sensitivity for the detection of cystic lesions^7^. In recent studies based on ultrasound and computed tomography and in abdominal explorations simple hepatic cysts occurred in 2.5–18% of patient examinations^7–10^. In the majority of cases, cystic hepatic lesions are detected incidentally in clinical imaging. Yet, only little is known about the change in the size of benign cystic hepatic lesions in healthy individuals.

Also, risk factors and clinical association for the occurrence of liver cysts are discussed. For example, authors demonstrated an association between simple hepatic cysts and age^7–11^. In addition, the prognostic factors for the development of cystic hepatic lesions are unknown.

Fortunately, only a few of the incidentally detected cysts are of malignant origin. Malignant cystic hepatic lesions account for less than 5% of all cystic hepatic lesions^5^. The most important differential diagnoses are cystic adenocarcinomas and cystic metastases. Typically, cystic metastases can originate from colonic adenocarcinoma, melanoma, carcinoids as well as breast-, renal- or ovarian cancer. Their cystic appearance can be explained by insufficient arterial blood supply causing an extensive central necrosis due to their rapid growth^12^, a striking difference to benign hepatic lesions. Especially in patients with a tumor in their medical history, characterization of cystic liver lesions by imaging is always a challenge^12^. Some authors discussed extensive growth of cystic liver lesions as an indicator for malignancy^13,14^. However, few authors reported growth of simple hepatic cysts^15^ and little is known about the growth rate of simple benign cystic lesions. Therefore, the aim of our study was to investigate the frequency and risk factors of incidentally detected liver cysts in the general population in a longitudinal survey using MRI. Additionally, we determined changes in cyst size between baseline and 5-years follow-up to define physiological growth rate of simple hepatic cysts.

## Material and methods

Volunteers were recruited from the database of the Study of Health in Pomerania (SHIP)^16^. SHIP is a population-based study founded in 1997 in the northeast of Germany. SHIP was started with the first cohort SHIP-0 including 4.308 participants who were examined from 1997 to 2001. The same cohort was continuously followed up between 2002 and 2006 (SHIP-1, 3.300 participants), then 2008–2012 (SHIP-2, 2.333 participants), and 2013–2017 (SHIP-3, 1.718 participants). Starting in SHIP-2, a whole-body MRI was performed including strong T2-weighted sequences of the liver. The identical MRI protocol was repeated for the same volunteers examined in SHIP-3 after 5 years. SHIP was approved by the Ethics Committee of the University of Greifswald and conforms to the Declaration of Helsinki. Written informed consent was obtained from all participants.

### Study population

In this project, we included 656 volunteers who participated at the MRI examination in SHIP-2 and at the 5-years follow-up (SHIP-3). We excluded 49 participants who reported a medical history of a malignant tumor resulting in a study population of 607 individuals.

### MRI examination

Whole-body MRI was performed using a standardized sequence protocol in all volunteers on a 1.5 T MRI scanner (Magnetom, Avanto; Siemens Healthcare, Erlangen, Germany) using integrated coil elements and phased-array surface coils (10). For the detection of cystic hepatic lesions, axial T2-weighted 2D TSE (Turbo-Spin-Echo) sequences were acquired using the following sequence parameters: TR: 8110 ms; TE: 116 ms; flip angle: 150°; number of averages: 1; bandwidth: 500 Hz/pixel; matrix size: 256 × 256; slices 32; slice thickness 6 mm. To reduce artefacts images were generated in BLADE (proprietary name for periodically rotated overlapping parallel lines with enhanced reconstruction) technique. To evaluate a coexistence of parenchymal liver diseases parametrical maps such as proton density fat fraction and R2* mapping (as expression of fatty liver diseases, liver iron overload, respectively) were acquired. For this reason, a three-echo 3D GRE (gradient echo) sequence was acquired for each participant at baseline. Sequence details are TR/TE1/TE2/TE3: 11/2.4/4.8/9.6 ms; flip angle: 10°; number of signal averages: 1; bandwidth: 1065 Hz/pixel; field of view: 410 × 308 mm; matrix size: 224 × 168 × 64; parallel imaging (GRAPPA) with effective acceleration factor of 1.8. Images were acquired in the axial orientation during a single 19-s breath-hold. Details of the post-processing for generating PDFF and R2* maps are described elsewhere^17^.

### Data analysis

A simple liver cyst was defined as a sharp bordered lesion with a bright signal compared to healthy liver and splenic tissue. Any lesion not fulfilling these criteria was omitted. We assessed all visible cystic lesions with a diameter of at least 2 mm. One trained observer (medical student) reviewed all images with regard to the occurrence and the frequency of liver cysts, at baseline and 5-years follow-up. In addition, the size of the largest cyst was determined. The observer was blinded to demographic and clinical data. The presence of cysts, the number, and their size were recorded. Image analysis was performed using the DICOM image viewer Horos (Horos Project version 3.3.6; https://horosproject.org). For quality assurance, the presence of simple liver cysts was assessed in a second reading by observer one by evaluating 98 participants of the baseline examination (15% of all cases). Additionally, the same sub-sample was reviewed by a second advanced observer with 15 years’ experience in abdominal MRI, main focus liver MRI.

### Risk factors and associations

Results of the baseline examination were compared to demographics, laboratory findings associated with liver diseases, and information about parenchymal liver disease as well as metabolic syndrome. The participant’s demographics, such as age, gender, waist-to-hip ratio, systolic blood pressure, diastolic blood pressure, and body mass index (BMI) were collected. Additionally, the following serum parameters were assessed: alanine transaminase (ALT), aspartate transaminase (AST), gamma-glutamyltransferase (GGT), partial thromboplastin time (PTT), prothrombin time (quick´s time), albumin, amylase, ferritin, choline esterase, bilirubin (direct/total), total cholesterol, HDL-cholesterol, LDL-cholesterol, triglycerides, glucose, and uric acid. In addition, we correlated the data with liver volume, and with the presence of parenchymal liver disease based on parametric MRI data such as liver iron overload (R2*) and fatty liver diseases (PDFF), respectively.

### Statistical analysis

Baseline characteristics of the study sample and nonparticipants were expressed by number and percentage for categorical variables and by median and interquartile range for continuous variables. Interobserver and intraobserver variability were determined for the variable “presence of liver cyst” by a double reading of 15% of the cases by two observers. The frequency of cystic hepatic lesions was calculated for the whole cohort as well as separately for men and women. Differences in cystic hepatic lesion frequency between men and women were tested using the χ^2^-test. The association of age with cyst frequency was assessed by logistic regression and the resulting prevalence curve for hepatic cysts was plotted against age. The numbers and sizes of cystic hepatic lesions in the subset of participants with cystic hepatic lesions were summarized by mean and standard deviation for men and women and differences were tested by Wilcoxon rank-sum tests. Differences in cystic hepatic lesions number according to age were evaluated by negative binomial regressions and results are expressed as rate ratios (RR) and 95% confidence intervals (CI). The association between age and the size of the largest hepatic cyst was analysed by median regression. Stratified by occurrence of hepatic cysts markers from laboratory and MRI are reported by means and standard deviations (SD). Associations of hepatic cysts with these markers were evaluated by separate linear regression models adjusted for age and sex. In longitudinal analyses, the incidence of hepatic cysts was calculated. In the group of individuals with hepatic cysts at both time points changes in cyst number and maximal cyst size were expressed by mean and SD. Associations of sex and age at baseline were associated with cyst incidence by Poisson regression and results are expressed as incidence rate ratio (IRR) and 95% CI’s. In individuals having cysts at both time points sex and age at baseline were associated with cyst number and size by linear regression models. A value of *p* < 0.05 was considered statistically significant. Statistical analysis was performed using Stata 16.0 (Stata Corporation, College Station, TX, USA).

## Results

### Inter-rater reliability and intra-rater reliability

Double reading of 98 participants revealed an excellent intra-rater reliability (IRR = 1) in relation to cyst occurrence. Regarding the cyst number the inter-rater variability showed an average difference of 0.06 ± 0.77.

### Frequency of hepatic cysts

**Figure 1:**
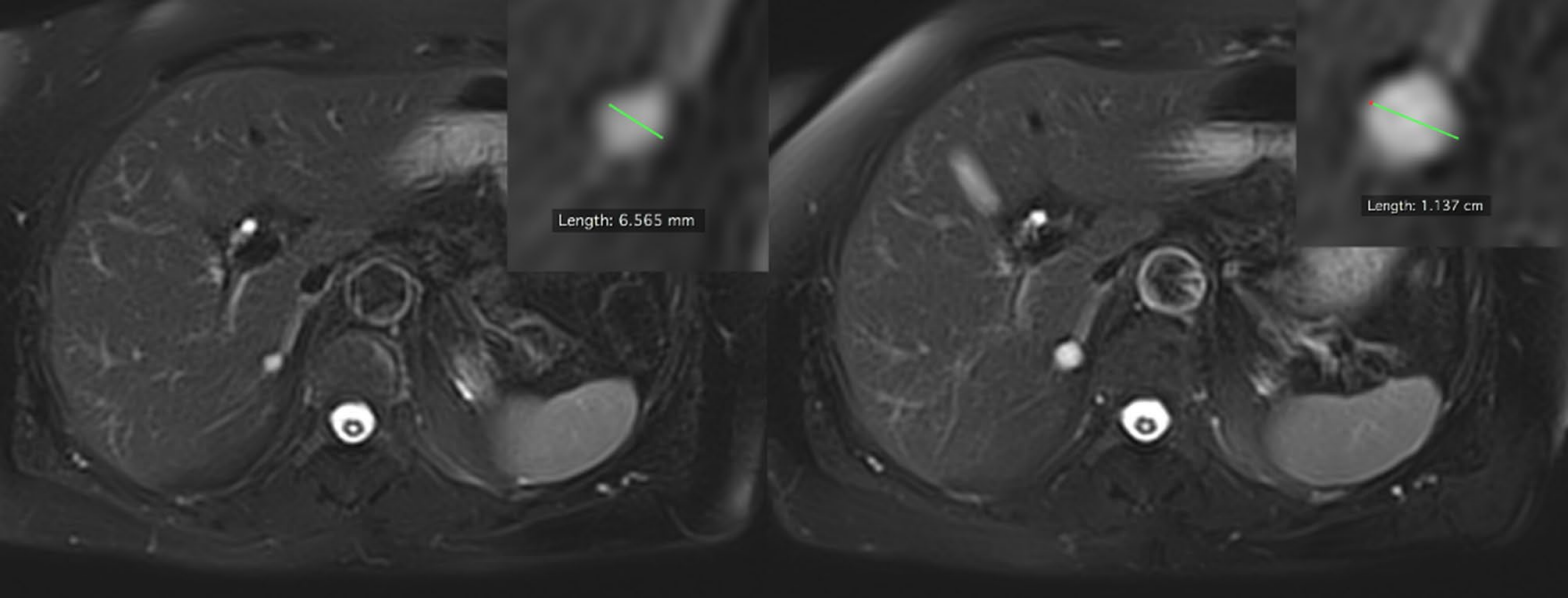
Case with a cyst at baseline and follow-up in segment 6 of the liver on axial T2-weighted TSE sequence. The enlarged segments in the right upper corner of both images depict an example of a cyst measurement. Its size changed from 6.6 mm to 11.4 mm.

**Figure 2:**
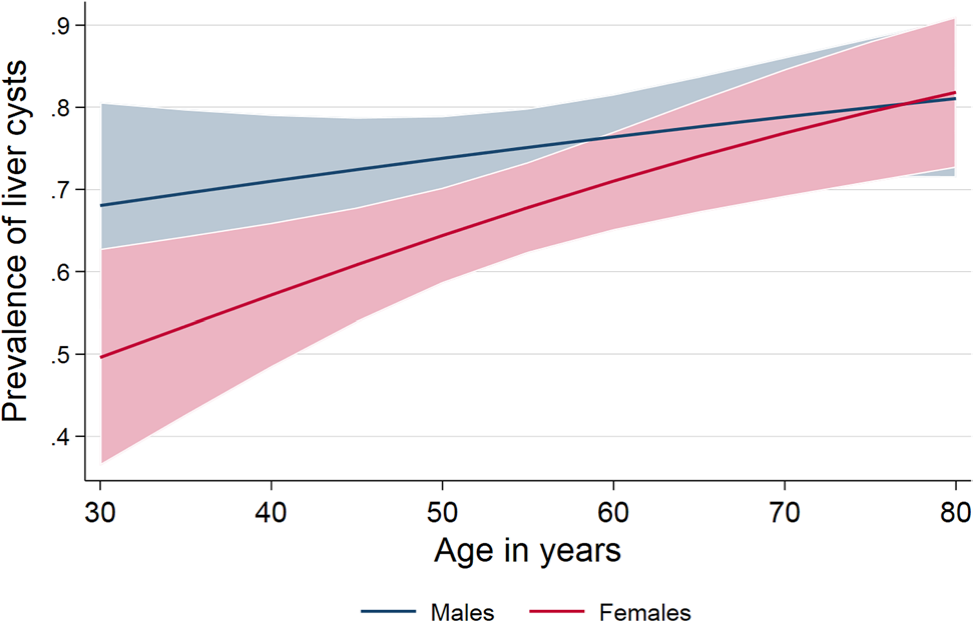
Association between age and cyst frequency for male and female volunteers.

The final analytical sample included 607 participants consisting of 288 women, 319 men, with a mean age of 54.4 ± 12.1 years. Four hundred thirty-one volunteers showed a total of 1,479 hepatic cysts at baseline. The frequency of liver cysts in our study population was 71.0%. Cyst frequency was significantly lower in females (n = 192; 66.7%) than in males (n = 239; 74.9%; *p* = 0.025). Figure 1 demonstrates a case with cysts at baseline and follow-up. In the whole study population, older volunteers demonstrated more often cysts than younger ones (OR: 1.02 per year of age; 95th CI 1.01–1.04; *p* = 0.002) (Fig. 2). The highest cyst frequency (81.8%) was seen in subjects of 80 years and older and the lowest frequency was seen at the age between 30 and 40 years (61.3%). In contrast to the female population (OR: 1.03 per year of age; 95th CI 1.01–1.05; *p* = 0.004), age was not significantly associated with cyst frequency in men (OR: 1.01 per year of age; 95th CI 0.99–1.04; *p* = 0.210).

### Number of cysts

**Figure 3:**
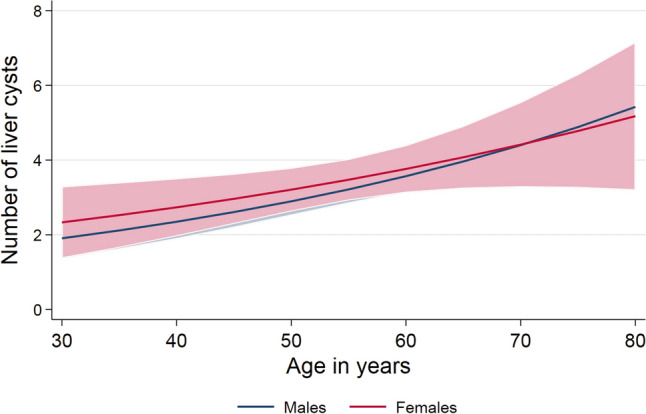
Association between mean number of cysts and age for male and female volunteers.

Of the subjects with cysts 88.6% (n = 382) had 1–5 cysts, 7.7% (n = 33) showed 6–10 cysts, 2.3% (n = 10) 11–20 cysts, and 1.4% (n = 6) had more than 20 cysts. The mean number of cysts in individuals having cysts was 3.4 ± 9.0. On average, women had significantly more cysts than men (3.6 ± 12.2 cysts vs. 3.3 ± 5.2, *p* = 0.026). In the whole population (RR: 1.019 (95th CI 1.010–1.027), *p* < 0.001) as well as divided by males (RR: 1.021 (95th CI 1.011–1.031), *p* < 0.001) and females (RR: 1.016 (95th CI 1.001–1.031), *p* = 0.032), age was significantly associated with cyst number (Fig. 3).

### Cyst size

**Figure 4:**
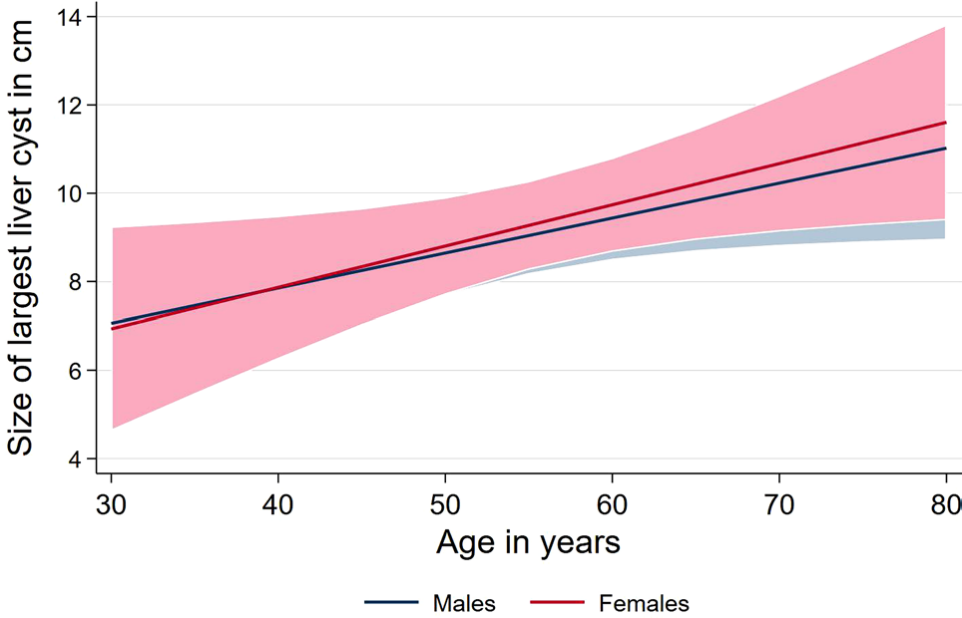
Association between mean size of cysts and age for male and female volunteers.

The intraindividual maximum cyst size was measured < 5 mm in 0.2% (n = 1) of all subjects. Cysts 5.1–10.0 mm were found in 53.4% (n = 230), and > 10.1 mm in 46.4% (n = 200). The mean cyst diameter was 13.1 ± 11.7 mm for the whole study sample. There was no significant difference in cyst size between male (13.6 ± 12.6 mm) and female participants (12.6 ± 10.4 mm), *p* = 0.625. A significant increase of the cyst size with age was observed for both genders (men, β: 0.08, 95th CI 0.01–0.16, *p* = 0.042; women β: 0.09, 95th CI 0.01–0.18, *p* = 0.025) (Fig. 4).

### Association of cyst occurrence with laboratory and imaging markers

Table 1 demonstrates an overview of examined potential associations of the occurrence of benign cystic hepatic lesions with selected markers from laboratory and MRI. After adjustment for age and gender, hepatic cysts were associated with a significant lower BMI, serum total protein, ALT and liver fat as determined by PDFF in MRI. Furthermore, hepatic cysts were significantly associated with lower triglygerides, lower glucose levels, less uric acid levels, a more favourable waist-to-hip ratio, and lower systolic blood pressure.

**Table 1:** Associations of selected laboratory markers from and MRI with liver cyst occurrence Data are expressed as mean and standard deviation; β’s were derived from linear regression models adjusted for age and sex with the respective marker as outcome and liver cyst occurrence as exposure. Significant results are given in bold letters. CI = confidence interval; *p < 0.05; PTT – partial thromboplastin time; ALT – alanine transaminase; AST – aspartate transaminase; GGT – gamma-glutamyltransferase.

	Liver cysts		β (95% CI)
	No (n = 176)	Yes (n = 431)	
Body mass index; kg/m^2^	**28.3 (4.5)**	**27.0 (4.0)**	− **1.43 (**− **2.16; **− 0.71)*
PTT; s	25.9 (3.1)	26.0 (2.8)	0.18 (− 0.33; 0.69)
Quick; %	106 (16)	107 (16)	0.53 (− 2.24; 3.29)
Serum albumin; g/L	40.5 (2.7)	40.2 (2.8)	− 0.19 (− 0.68; 0.30)
α-Amylase; µkatal/L	0.91 (0.30)	0.96 (0.35)	0.04 (− 0.02; 0.10)
Ferritin; µg/L	154 (210)	128 (128)	− 24.0 (− 50.4; 2.4)
Transferrin; g/L	2.60 (0.40)	2.54 (0.41)	− 0.06 (− 0.13; 0.02)
Lipase; µkatal/L	2.84 (1.44)	2.69 (0.95)	− 0.19 (− 0.39; 0.01)
Beta2-Microglobulin; µmol/L	1.72 (0.40)	1.82 (0.93)	0.04 (− 0.10; 0.18)
Cholinesterase; µkatal/L	212 (41)	206 (39)	− 6.38 (− 13.29; 0.53)
Total protein; g/L	**72.3 (3.9)**	**71.3 (4.4)**	− **0.87 (**− **1.64; **− 0.11)*
Direct bilirubin; µmol/L	2.10 (0.54)	2.19 (0.67)	0.11 (− 0.01; 0.22)
Total bilirubin; µmol/L	7.94 (3.23)	8.56 (3.98)	0.69 (0.03; 1.36)
ALT; µkatal/L	**0.50 (0.30)**	**0.43 (0.26)**	− **0.05 (**− **0.10; **− 0.01)*
AST; µkatal/L	0.35 (0.17)	0.33 (0.17)	− 0.03 (− 0.06; 0.01)
GGT; µkatal/L	0.76 (0.89)	0.66 (0.69)	− 0.09 (− 0.21; 0.43)
Liver fat (PDFF); %	**8.27 (8.21)**	**6.04 (5.75)**	− **2.36 (**− **3.68; **− 1.05)*
Liver iron (R2)	39.0 (20.9)	37.9 (18.7)	− 0.96 (− 4.83; 2.91)
Fibrosis score	0.99 (0.57)	1.07 (0.65)	− 0.01 (− 0.10; 0.07)
Total cholesterol; mmol/L	5.50 (0.99)	5.56 (1.04)	− 0.01 (− 0.19; 0.17)
HDL-cholesterol; mmol/L	1.44 (0.43)	1.51 (0.43)	0.04 (− 0.03; 0.11)
LDL-cholesterol; mmol/L	3.21 (0.83)	3.26 (0.84)	0.02 (− 0.13; 0.17)
Triglycerides; mmol/L	1.88 (1.29)	1.66 (0.93)	− **0.23 (**− **0.41; **− 0.05)*
Glucose; mmol/L	5.78 (1.19)	5.50 (1.04)	− **0.30 (**− **0.48; **− 0.11)*
Uric acid; µmol/L	290 (70)	268 (72)	− **16.9 (**− **27.2; **− 6.6)*
Waist to hip ratio	0.90 (0.08)	0.88 (0.09)	− **0.011 (**− **0.021; **− 0.001)*
Systolic blood pressure; mmHg	133 (18)	130 (18)	− **3.99 (**− **6.84; **− 1.14)*
Diastolic blood pressure; mmHg	82 (10)	80 (10)	− 1.47 (− 3.16; 0.22)

### Association of cyst number and size with laboratory and imaging markers

An inverse association of the liver cysts’ size with BMI, transferrin, protein serum levels, percental liver fat (PDFF), as well as uric acid levels, was found (Table 2). No additional association of cyst number and laboratory markers was detected.

**Table 2:** Associations of liver cyst number and size with selected markers from laboratory and MRI; β’s are derived from linear regression models adjusted for age and sex with the respective marker as outcome and liver cyst number and size as exposures. Significant results are given in bold letters. CI = confidence interval; *p < 0.05; PTT – partial thromboplastin time; ALT – alanine transaminase; AST – aspartate transaminase; GGT – gamma-glutamyltransferase.

	Liver cyst number β (95% CI)	Liver cyst size β (95% CI)
Body mass index; kg/m^2^	0.008 (− 0.034; 0.051)	− **0.031 (**− **0.060; **− 0.001)*
PTT; s	0.011 (− 0.019; 0.041)	0.003 (− 0.017; 0.023)
Quick; %	− 0.036 (− 0.197; 0.124)	0.050 (− 0.059; 0.159)
Serum albumin; g/L	− 0.008 (− 0.037; 0.020)	− 0.008 (− 0.028; 0.011)
α-Amylase; µkatal/L	− 0.000 (− 0.004; 0.003)	0.000 (− 0.001; 0.003)
Ferritin; µg/L	− 0.820 (− 2.337; 0.698)	− 0.844 (− 1.877; 0.190)
Transferrin; g/L	0.001 (− 0.003; 0.005)	− **0.003 (**− **0.006; **− 0.000)*
Lipase; µkatal/L	0.001 (− 0.010; 0.013)	− 0.006 (− 0.014; 0.001)
Beta2-Microglobulin; µmol/L	− 0.001 (− 0.009; 0.007)	0.001 (− 0.004; 0.007)
Cholinesterase; µkatal/L	− 0.298 (− 0.697; 0.100)	− 0.040 (− 0.312; 0.232)
Total protein; g/L	− 0.022 (− 0.066; 0.022)	− **0.035 (**− **0.065; **− 0.005)*
Direct bilirubin; µmol/L	0.002 (− 0.004; 0.009)	0.002 (− 0.003; 0.006)
Total bilirubin; µmol/L	0.010 (− 0.029; 0.048)	0.014 (− 0.012; 0.040)
ALT; µkatal/L	0.002 (− 0.001; 0.005)	− 0.001 (− 0.003; 0.001)
AST; µkatal/L	0.002 (− 0.001; 0.003)	− 0.001 (− 0.002; 0.001)
GGT; µkatal/L	0.005 (− 0.003; 0.012)	− 0.004 (− 0.009; 0.001)
Liver fat (PDFF); %	− 0.020 (− 0.090; 0.050)	− **0.076 (**− **0.132; **− 0.020)*
Liver iron (R2)	− 0.081 (− 0.285; 0.122)	− 0.095 (− 0.258; 0.068)
Fibrosis score	0.000 (− 0.004; 0.005)	− 0.000 (− 0.004; 0.003)
Total cholesterol; mmol/L	− 0.003 (− 0.014; 0.007)	0.003 (− 0.004; 0.010)
HDL-cholesterol; mmol/L	0.004 (− 0.001; 0.008)	0.001 (− 0.002; 0.003)
LDL-cholesterol; mmol/L	− 0.005 (− 0.013; 0.004)	0.004 (− 0.002; 0.010)
Triglycerides; mmol/L	− 0.009 (− 0.020; 0.001)	− 0.006 (− 0.013; 0.001)
Glucose; mmol/L	− 0.006 (− 0.016; 0.005)	− 0.006 (− 0.013; 0.001)
Uric acid; µmol/L	− 0.39 (− 0.99; 0.20)	− **0.61 (**− **1.02; **− 0.21)*
Waist to hip ratio	− 0.000 (− 0.001; 0.001)	− 0.000 (− 0.001; 0.001)
Systolic blood pressure; mmHg	0.18 (0.01; 0.34)*	− 0.07 (− 0.18; 0.04)
Diastolic blood pressure; mmHg	0.08 (− 0.02; 0.17)	− 0.00 (− 0.07; 0.06)

### Frequency of liver cysts

Over a median follow-up time of 4.8 years (SD 0.8), 381 (88.4%) of 431 individuals with hepatic cysts at baseline also had hepatic cysts at follow-up. 25.1% of the subjects (n = 36) with no initial liver cyst showed new lesions. Hepatic cysts disappeared after 5 years in 11.6% (50 cases) of all subjects with cysts at baseline. For cyst incidence no significant differences were observed between both sexes (IRR = 1.20; 95% CI 0.62 to 2.31; *p* = 0.539) or with age at baseline (IRR = 1.00; 95% CI 0.97 to 1.03; *p* = 0.990).

Individuals with disappearing cysts during follow-up had a mean number of 1.3 (SD 0.7) cysts at baseline. Individuals with no initial cysts had a mean cyst count of 1.8 (SD 1.1) at follow-up. For individuals with cysts at both time points, the mean number increased from 3.7 (SD 9.5) to 4.5 (SD 10.9) cysts during follow-up (Table 3). Overall, this increase over time was significant (*p* < 0.001) but did not differ significantly according to sex (β = − 0.11; 95% CI − 0.88 to 0.65; *p* = 0.264) or age (β = − 0.00; 95% CI − 0.03 to 0.03; *p* = 0.833).

**Table 3:** Mean number of cysts during both time points; SD = standard deviation.

	Overall Mean ± SD	Men Mean ± SD	Women Mean ± SD
Baseline	3.7 ± 9.5	3.6 ± 5.4	3.8 ± 13.1
Persistent in follow-up	4.5 ± 10.9	4.3 ± 6.8	4.7 ± 15.4
New	1.8 ± 1.1	1.7 ± 1.0	1.9 ± 1.1
Disappeared	1.3 ± 0.7	1.2 ± 0.5	1.5 ± 0.8

Individuals with newly developed cysts reached a maximum cyst size of 6.9 mm (SD 1.8) during follow-up, whereas in the case of disappearing cysts, the maximum size at baseline was 8.8 mm (SD 8.0). For individuals with cysts at both time points, the maximum cyst size increased on average from 13.7 mm (SD 11.9) to 15.3 mm (SD 12.1) during follow-up (Table 4). Overall, this increase over time was significant (*p* < 0.001) but did not differ significantly according to gender (β = 0.20; 95% CI − 0.10 to 0.06; *p* = 0.593) or age (β = − 0.02; 95% CI − 0.10 to 0.06; *p* = 0.593).

**Table 4:** Mean maximum cyst size (mm) during both time points; SD = standard deviation.

	Overall Mean ± SD	Men Mean ± SD	Women Mean ± SD
Baseline	13.7 ± 11.6	14.2 ± 12.9	13.1 ± 10.6
Follow-up	15.3 ± 12.1	15.9 ± 11.6	14.6 ± 12.6

## Discussion

In our study, we investigate the frequency, incidence, and risk factors of liver cysts in a healthy population in a longitudinal survey. With a frequency of 71%, cystic liver lesions are a common incidental finding. Besides associations with age and sex, there is an inverse relation with factors of the metabolic syndrome. Progression of the number of cysts and the increased size are a physiological phenomenon in long term follow-up.

Contradictory to results of previous studies^7–10^, liver cysts are a common finding in healthy individuals (71% vs. 0.17–18%). This large difference between recent studies and our results is explained by the high sensitivity of T2-weighted MRI for the detection of fluid. Liver cysts are an abundant finding on MRI because it combines all the advantages of the techniques mentioned above. It is observer-independent and provides high sensitivity for cysts at the same time. Therefore, liver cysts can be counted among “disease of technology” owing to the ever-increasing advances in spatial resolution and signal of MRI. Based on computed tomography, Carrim et al. found a cyst frequency of 18%^9^. Gaines et al. found a frequency of 2.5% in a large general population using ultrasound^8^, and Kaltenbach et al. found cysts in 5.8% of ultrasound reports of a large population of hospital patients^7^. These data emphasize that despite the high sensitivity for cysts, ultrasound is observer-dependent, and especially small cysts presumably could be missed. Sanfelippo et al. recorded a frequency of 0.17%^10^ from a large series of abdominal explorations of symptomatic patients. It is highly doubtful that abdominal explorations accurately depict smaller cysts, as already put into perspective in their work.

According to results from abdominal explorations and imaging-based studies, the overall frequency of liver cysts rises with age^10^ which is confirmed with our observation. In this study, most of the volunteers had less than 5 cysts (88.6%). Kaltenbach et al. found up to 5 cysts in 62.8% of the cases^7^. Sanfelippo et al. reported solitary cysts in 74.5% and Gaines et al. in 74%^8,10^. The latter did not elaborate on the number of cysts more closely. Our study cohort was markedly younger than the ones of the studies mentioned above with an average age of 54.4 years (vs. 59–65 years). Bearing in mind that cyst frequency rises with age, we ascribe the higher percentage of solitary cysts in our cohort to the high sensitivity of strong T2-weighted sequences for fluid. Concurring with other studies, a predominance of cysts in women was noted^10,15^. Whilst the other study groups found average cyst sizes between 1.2 and 22.3 mm^7,8,10^, our cohort displayed average diameters of 13.1 mm. Based on our findings, cyst size rises with age. Accordingly, the expected average cyst size should be smaller than the results of previous studies obtained with older patients. These differences might originate from the selected patient population and the fact that we observed healthy individuals.

So far, no study has analysed the association between the occurrence of liver cysts and clinical parameters such as demographics, laboratory data and factors associated with the metabolic syndrome. Volunteers without cysts had a higher BMI and a higher percental liver fat. Also, the inverse association of the liver cysts’ size with BMI correlates with lower liver fat measured on fat fraction on MRI. From these first findings we hypothesized that individuals without cysts might show more features of metabolic syndrome. Main criteria for this diagnosis are fasting glucose ≥ 100 mg/dL (5,6 mmol/L) or type 2 diabetes; blood pressure ≥ 130 mm Hg; triglycerides ≥ 150 mg/dL (1,7 mmol/L); HDL-C < 40 mg/dL (< 1.03 mmol/L) in men and < 50 mg/dL (1,29 mmol/L) in women and waist circumference of ≥ 102 cm in men and ≥ 88 cm in women^18^. In this healthy population these pathological laboratory results and higher waist to hip ratios, indicating central obesity, were found in volunteers without cysts. These individuals also had higher serum levels of uric acid, a minor criterion for metabolic syndrome. In contrast, individuals with cysts stayed within the physiological range of the laboratory values mentioned above. Therefore, there is an inversed relation with cyst occurrence and size, which might reflect that cysts belong to the phenotypic spectrum of metabolic syndrome^19^. As only healthy volunteers were included in this survey, more studies with individuals suffering metabolic syndrome and liver disease are needed to verify this possible coherence.

Results of genetic and immunohistochemical studies on solitary simple cysts, multiple liver cysts and cysts in polycystic liver disease (PLD) implicate the presence of a continuous spectrum of these entities^20–24^. We therefore searched for analogies in laboratory findings of PLD patients and the part of our cohort with cysts. PLD patients have elevated levels of GGT^25,26^, AST (27%), total bilirubin (15%), and ALT^26^. Interestingly, in this study the volunteers with cysts presented tendentially lower GGT levels than the patients without cysts. Likewise, total, as well as direct bilirubin did not differ between both groups. Both, protein and ALT were higher in individuals without cysts or smaller cysts (here only protein) countering the findings made in PLD patients.

In our study, 20.5% of participants developed liver cysts within a five years follow-up. To this date, no study has investigated the average intraindividual growth of liver cysts in a healthy population. According to the findings of this study, the chance of developing a cyst rises by 2% per year of age. Overall, both average cyst size and cyst number increase over time. Especially participants with a higher maximum cyst size and number appeared to belong to this group. In contrast, individuals with a relatively small maximum cyst size showed cyst regression. Hence, this might serve as a prognostic factor to assess the development of cysts over time. As much as 11.6% showed a spontaneous regression during follow-up. This finding goes along with a series of 31 children with antenatally detected unilocular cysts showing regression as well as persistence in the follow-up^15^. Nevertheless, in adults, cyst regression has only been reported in a single case up to now^27^. Although the detection of extremely small cysts is limited by the slice thickness of the MRI we interpret the finding of cyst regression as reliable because the cysts had been detectable in the baseline survey.

None of the individuals developed a malignant liver tumor. Taken together with histopathological findings of simple liver cysts^28^ the risk for a malignant transformation is very low. However, histopathological findings of autopsied livers show an expression of apomucin MUC1 in the epithelial lining of 71–100% of “normal” liver cysts, which is frequently expressed in cholangiocarcinoma^20^. Thus, the risk for a malignant transformation cannot be estimated from our observation after this short period of time. Studies with longer follow-ups are needed to estimate this risk.

Our study has some limitations. Firstly, it is an observational study with a high risk of type 1 error. Secondly, very small cysts might be overseen due to the slice thickness of 6 mm. As can be seen in our data, the diameter of the detected cysts was more than 5 mm in the majority of cases. Thirdly, the strong T2-weighted images are not suitable for a dedicated analysis of the cyst wall or solid components. Furthermore, the diagnosed cysts were not confirmed by a second imaging technique or by histology. For example, by using the strong T2 weighted MRI alone, we cannot differentiate between simple cysts and liver hemangioma.

In conclusion, cystic liver lesions are a common incidental finding in the general population. Higher age and male gender are associated with the occurrence of liver cysts. Cystic lesion progression and the increased size are physiological phenomena in long term follow-up. Cysts were predominantly detected in a healthier population and might bear the potential as an imaging marker for healthy liver tissue.

## Abbreviations

ALAT: Alanine transaminase
ASAT: Aspartate transaminase
BMI: Body mass index
CI: Confidence interval
CT: Computed tomography
GGT: Gamma-glutamyltransferase
IRR: Incidence rate ratio
MRI: Magnetic resonance imaging
PTT: Partial thromboplastin time
RR: Rate ratio
SD: Standard deviation
SHIP: Study of Health in Pomerania
TSE: Turbo-Spin-Echo
